# S100 as marker for immune effector cell-associated neurotoxicity syndrome

**DOI:** 10.1007/s00508-024-02451-0

**Published:** 2024-10-04

**Authors:** Axel Schulenburg, Lina Z. Rüsing, Armin Bumberger, Margit Mitterbauer, Werner Rabitsch

**Affiliations:** 1 Bone Marrow Transplantation Unit, Department of Internal Medicine I, Medical University of Vienna, Vienna, Austria; 2 Department of Medicine I, Stem Cell Transplantation Unit, Medical University of Vienna, Waehringer Guertel 18–20, 1090 Vienna, Austria

**Keywords:** Chimeric antigen receptor (CAR)-T cell therapy, Side effects, Toxicity, Marker, Neurology

## Abstract

Chimeric antigen receptor (CAR)-T cell therapy is a new and successful treatment for otherwise refractory malignancies but despite the growing number of applications, this form of treatment is still associated with significant toxicity. Cytokine release syndrome (CRS) and immune effector cell-associated neurotoxicity syndrome (ICANS) in particular are common and dangerous side effects. This report is about two patients who received CAR‑T cell therapy and subsequently developed ICANS. This was successfully treated. During CAR‑T cell therapy, a blood marker, S100, was monitored daily. It correlated with the occurrence and progression of ICANS.

## Introduction

Chimeric antigen receptor (CAR)-T cell therapy is a groundbreaking form of cancer treatment that involves reprogramming a patient’s T cells to recognize and attack cancer cells. This personalized immunotherapy has shown remarkable success in treating certain types of blood cancers, such as leukemia and lymphoma [1–3]; however, one of the potential side effects of CAR‑T cell therapy is the development of a condition called immune effector cell-associated neurotoxicity syndrome (ICANS) [4]. This can cause symptoms such as confusion, delirium, seizures, and other neurological issues. While the exact cause of ICANS is not fully understood, it is believed to be related to the immune response triggered by the CAR‑T cells, leading to inflammation in the brain. Medical professionals closely monitor patients undergoing CAR‑T cell therapy for signs of ICANS and have developed protocols to manage and treat this potential side effect [5, 6]. Research in this area is ongoing to better understand, prevent, and treat ICANS, allowing for the continued advancement of CAR‑T cell therapy as a promising cancer treatment.

Therefore, it would be highly desirable if there were a factor that correlates with the occurrence of ICANS. This could help predict which patients are at high risk of developing this side effect. It could also be useful in tailoring ICANS therapy. In this context, we came across a marker called S100. The blood marker S100 is used to diagnose and monitor the progression of certain diseases. It refers to a group of proteins that occur in various tissues of the body, including the nervous system. The S100 proteins can be measured in blood tests and serve as indicators of various conditions. The importance of S100 lies mainly in neurology and oncology. In neurology, an increased S100 value can indicate damage to the brain or spinal cord, for example, after stroke, injury, or neurodegenerative disease. In oncology, the S100 value can be helpful in diagnosing and monitoring melanomas and other types of cancer.

Of particular interest to us was that S100 is also a marker for a damaged blood-brain barrier [7]. In addition to their diagnostic significance, S100 proteins play various biological roles. They are calcium-binding proteins that modulate cellular functions such as proliferation, differentiation, and apoptosis [8]. They act as inflammatory mediators, participate in interactions between neurons and glial cells, serve as markers for tissue damage, and indicate the integrity of the blood-brain barrier [9]. Understanding these functions can further clarify the role of S100 in diagnostics and treatment. To evaluate the correlation of this marker with ICANS, we monitored it daily in a series of patients who underwent CAR‑T cell therapy at our institution.

## Case report 1

**Fig. 1:**
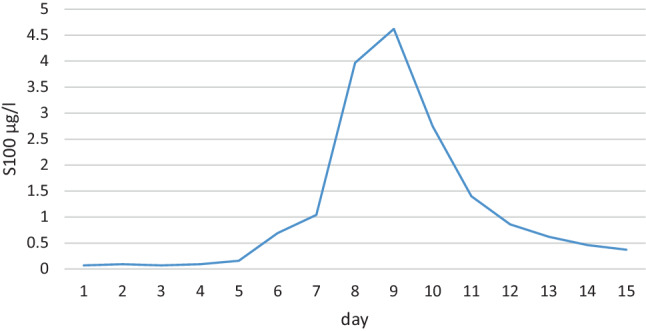
S100 over time for patient 1. Normal range: S100 < 0.105 µg/l

A 67-year-old male was diagnosed with mantle cell lymphoma stage IVb. He received treatment with the R‑CHOP (rituximab, cyclophosphamide, doxorubicin, vincristine, and prednisone) regimen for 6 cycles followed by maintenance therapy with rituximab. Unfortunately, he relapsed after 12 months. Subsequently, the therapy was switched to imbruvica. After 4 months he progressed again with non-bulky generalized lymphadenopathy without B symptoms. Based on this finding, he was referred to our institution to receive CAR‑T cell therapy. He underwent successful leukapheresis. The CAR‑T cell therapy (Tecartus, Gilead Sciences, Munich, Germany) was administered following a lymphodepleting regimen with fludarabine 30 mg/m^2^ and cyclophosphamide 500 mg/m^2^ on days −5 to −3 before the infusion of Tecartus. On day +3 after CAR-T cell therapy the patient developed fever of 38.5 °C, hypoxia requiring 3 l/min of oxygen via nasal cannula and hypotension with need for vasopressor therapy on day +5 consistent with cytokine release syndrome (CRS) grades 2–3. After administration of tocilizumab (8 mg/kg x 4 times) the symptoms of CRS improved; however, since day +4 the level of S100 in the blood increased continuously (Fig. 1) and later on day +7 the patient developed ICANS with a decreased level of consciousness (Glasgow score 7–8), aphasia, and an immune effector cell-associated encephalopathy (ICE) score of 0. The magnetic resonance scan of the brain on the same day and the computed tomography of the brain on day +8 showed no intracranial abnormality. The electroencephalogram of day +8 showed alpha/theta background activity, signs of diffuse brain dysfunction (background activity, intermittent generalized delta slowing), no focal findings, no signs of increased cerebral excitability, and vigilance fluctuations. The patient was transferred on day +8 to the intensive care unit (ICU) for monitoring to be ready if intubation was necessary. Right from the beginning of ICANS on day +7 the patient received antiepileptic medication in form of levetiracetam combined with treatment with intravenous (IV) dexamethasone 10 mg every 6 h. At a peak blood level of S100 of 4.62µg/l and a lack of response to treatment on day +9 the medication was switched from dexamethasone to methylprednisolone 500mg every 12 h and anakinra at a dose of 200mg, subcutaneously, 3 times daily. Under this treatment the symptoms of ICANS improved and were completely resolved until day +13. This was accompanied by a steady decline of S100 blood levels. Therefore, the patient was transferred out of the ICU on day +11 and from that day on methylprednisolone and anakinra could be tapered and eventually discontinued on day +16, the day of discharge.

## Case report 2

**Fig. 2:**
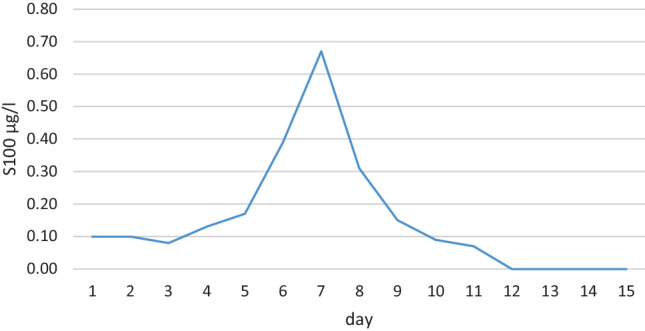
S100 over time for patient 2. Normal range: S100 < 0.105 µg/l

A 57-year-old male was diagnosed with acute lymphoblastic leukemia. He suffered from a relapse 11 months after allogenic blood stem cell transplantation. Therefore, he was referred to our institution to receive CAR-T cell therapy. He underwent successful leukapheresis. After that he received a bridging therapy of 1 cycle of inotuzumab ozogamicin. Subsequently, CAR-T cell therapy (Tecartus) was administered following a lymphodepleting regimen with fludarabine 25 mg/m^2^ on day −4 to −2 and cyclophosphamide 900 mg/m^2^ on day −2. On day +4 after CAR-T cell therapy the patient developed fever of 38.5 °C and hypoxia requiring 3 l/min of oxygen via nasal cannula consistent with CRS grade 1–2. After administration of tocilizumab (8 mg/kg x 4 times) the symptoms of CRS improved but since day +3 the level of S100 in the blood increased continuously (Fig. 2) and later on day +6 at a peak blood level of S100 of 0.67 µg/l the patient presented with a depressed level of consciousness and an ICE score of 7–9 consistent with ICANS grade 2. The electroencephalogram of day +6 showed alpha-theta basic activity and theta slowdown, no focal findings, no signs of increased cerebral excitability and the computed tomography on the same day showed no abnormalities. Right from the beginning of ICANS on day +6 the patient received antiepileptic medication in form of levetiracetam combined with treatment with intravenous (IV) dexamethasone 10 mg every 6 h and anakinra at a dose of 200 mg, subcutaneously, 3 times daily. Under this therapy the symptoms of ICANS improved and were completely resolved until day +10. This was accompanied by a steady decline of S100 blood levels. Therefore, dexamethasone and anakinra could be tapered.

## Discussion

The ICANS is the most significant and dangerous side effect of CAR‑T cell therapy. Therefore, it is of great interest to find a way to predict this side effect or improve its management. To date, several methods have been attempted to predict the likelihood of ICANS [10, 11]. Patient characteristics, such as female sex, low level of platelets (< 150 G/L), use of Yescarta (Gilead Sciences, Munich, Germany) and no bridging therapy, have been used to classify patients into certain risk groups; however, a specific marker that reliably correlates with the onset of ICANS has not yet been identified. This may be due to factors such as the isolation of the central nervous system.

In our search for such a marker, we focused on S100, which is known to be a marker of blood-brain barrier dysfunction. We measured this marker in the blood of the patients daily, both before and after the administration of CAR‑T cells. The increase in S100 levels preceded the onset of clinical symptoms and also showed a strong correlation with the peak and subsequent response of ICANS to treatment. This could potentially help prevent ICANS in the future, or at least improve the adjustment of ICANS therapy; however, it remains speculative whether an increase in S100 truly indicates blood-brain barrier dysfunction following CAR‑T cell therapy.

In summary, to our knowledge, this is the first report of a marker that correlates with ICANS and is relatively easy to measure, as it only requires a blood sample. Nonetheless, further studies are needed to evaluate the significance of S100 in the context of ICANS.
